# INTERGENERATIONAL REMINISCENCE PROGRAM IN IMPROVING GENERATION CONNECTIONS: A RADAR ANALYSIS

**DOI:** 10.1093/geroni/igad104.2874

**Published:** 2023-12-21

**Authors:** Ling Xu, Minjaal Raval

**Affiliations:** The University of Texas at Arlington, Arlington, Texas, United States; The University of Texas at Arlington, Arlington, Texas, United States

## Abstract

**Objectives:**

Intergenerational solidarity between grandparents and grandchildren has been highlighted in Asian Families and it is beneficial for the health of grandparents. An intergenerational reminiscence program was developed to improve the well-being of Asian American older adults as well as generational bonding. This paper qualitatively reported the weekly reflection from grandchild participants of this program.

**Methods:**

Older grandparents received 6 sessions of life-review with their grandchildren remotely or in person for approximately 1 hour each week for 6 weeks. Each grandchild (n = 12) provided written reflection each week after talking with grandparent. The qualitative data were organized and analyzed using the five phases of the Rigorous and Accelerated Data Reduction (RADaR) technique.

**Results:**

Results showed three categories with themes. Category 1: Positive experience. The grandchildren expressed: (1) more connection with grandparent, (2) learn more about grandparent’s past life experience, and (3) have more engagement. Category 2: Strategy to lead the discussion. The grandchildren thought the following good ways in facilitating their weekly conversation with grandparents: (1) taking notes, (2) using guiding question in the manual; (3) use translators, (4) spending time together. Category 3: Challenging experience. The grandchildren felt challenges regarding: (1) over explaining things, (2) language or vocabulary barriers, (3) broad topic.

**Discussion:**

The reflection pages from grandchild participants showed that intergenerational reminiscence program is promising in bonding the grandparent-grandchild relationship and connection. They also gained knowledge and experienced challenges when talking with their grandparents during the program.

